# Fatty Acyl-CoA Reductase 1 Deficiency

**DOI:** 10.15844/pedneurbriefs-29-1-5

**Published:** 2015-01

**Authors:** Charles N. Swisher

**Affiliations:** 1 Division of Neurology, Ann & Robert H. Lurie Children's Hospital of Chicago, Chicago, IL; 2 Departments of Pediatrics and Neurology, Northwestern University Feinberg School of Medicine, Chicago, IL

**Keywords:** Peroxisomal disorder, Fatty Acyl-CoA Reductase 1, Developmental delay

## Abstract

Investigators from Erlangen, Germany; Calgary, CA; and Kafranbel, Syria, identified mutations in the gene, fatty acyl-CoA reductase 1 (FAR1) deficiency, adding to three other genes involved in plasmalogen biosynthesis, in two families affected by severe intellectual disability, early-onset epilepsy, microcephaly, congenital cataracts, growth retardation, and spasticity.

Investigators from Erlangen, Germany; Calgary, CA; and Kafranbel, Syria, identified mutations in the gene, fatty acyl-CoA reductase 1 (FAR1) deficiency, adding to three other genes involved in plasmalogen biosynthesis, in two families affected by severe intellectual disability, early-onset epilepsy, microcephaly, congenital cataracts, growth retardation, and spasticity. Exome analyses revealed a homozygous in-frame indel mutation in two siblings from a consanguineous family and compound-heterozygous mutations in a third unrelated individual. FAR1 reduces fatty acids to their respective fatty alcohols for the plasmalogen-biosynthesis pathway. All three mutations abolished the reductase activity of FAR1. The spectrum of clinical features associated with defects in plasmalogen biosynthesis is expanded to include FAR1 deficiency as a cause of syndromic severe intellectual disability with cataracts, epilepsy, and growth retardation but without rhizomelia. Mutations in PEX7, GNPAT, and AGPS are the genes previously described in individuals with rhizomelic chondrodysplasia punctata (RCDP). Defects in plasmalogen-biosynthesis lead to low levels of plasmalogens and to symptoms of RCDP. An increasing number of genetic disorders are recognized that result from defects in peroxisomal biogenesis (eg, Zellweger syndrome). [1]

COMMENTARY. We have come a long way from Garrod's “one gene, one enzyme” hypothesis regarding metabolic genetic disorders in 1909 and our present explorations of the ever-expanding geography and biochemical intricacy of the genome. Buchert et al. have expanded the etiological spectrum of one of the peroxisomal disorders, namely RCDP, which had formerly been associated with abnormalities in one of three genes to include the gene FAR1, which spares the skeletal system but is associated with severe intellectual disability seen in other forms. The teasing out of clinical findings based on the biochemical activity of specific genes is a quest for many geneticists today, and one elegantly demonstrated in this study. [2, 3]

## References

[1] Buchert R, Tawamie H, Smith C, Uebe S, Innes AM, Al Hallak B A peroxisomal disorder of severe intellectual disability, epilepsy, and cataracts due to fatty acyl-CoA reductase 1 deficiency. Am J Hum Genet..

[2] Roscher AA, Hoefler S, Hoefler G, Paschke E, Paltauf F, Moser A (1989). Genetic and phenotypic heterogeneity in disorders of peroxisome biogenesis--a complementation study involving cell lines from 19 patients. Pediatr Res..

[3] O'Rahilly S (2009). Human genetics illuminates the paths to metabolic disease. Nature.

